# Dipeptidyl-Aminopeptidases 8 and 9 Regulate Autophagy and Tamoxifen Response in Breast Cancer Cells

**DOI:** 10.3390/cells12162031

**Published:** 2023-08-10

**Authors:** Aaron Bettecken, Lisa Heß, Lena Hölzen, Thomas Reinheckel

**Affiliations:** 1 Institute of Molecular Medicine and Cell Research, Faculty of Medicine, University of Freiburg, 79104 Freiburg, Germany; 2 Faculty of Medicine, University of Freiburg, 79104 Freiburg, Germany; 3 Faculty of Biology, University of Freiburg, 79104 Freiburg, Germany; 4 German Cancer Consortium (DKTK), Partner Site Freiburg, 79104 Freiburg, Germany; 5 German Cancer Research Center (DKFZ), 69120 Heidelberg, Germany; 6 Centre of Biological Signalling Studies BIOSS, University of Freiburg, 79104 Freiburg, Germany

**Keywords:** DPP8, DPP9, breast cancer, autophagy, tamoxifen

## Abstract

The cytosolic dipeptidyl-aminopeptidases 8 (DPP8) and 9 (DPP9) belong to the DPPIV serine proteases with the unique characteristic of cleaving off a dipeptide post-proline from the *N*-termini of substrates. To study the role of DPP8 and DPP9 in breast cancer, MCF-7 cells (luminal A-type breast cancer) and MDA.MB-231 cells (basal-like breast cancer) were used. The inhibition of DPP8/9 by 1G244 increased the number of lysosomes in both cell lines. This phenotype was more pronounced in MCF-7 cells, in which we observed a separation of autophagosomes and lysosomes in the cytosol upon DPP8/9 inhibition. Likewise, the shRNA-mediated knockdown of either DPP8 or DPP9 induced autophagy and increased lysosomes. DPP8/9 inhibition as well as the knockdown of the DPPs reduced the cell survival and proliferation of MCF-7 cells. Additional treatment of MCF-7 cells with tamoxifen, a selective estrogen receptor modulator (SERM) used to treat patients with luminal breast tumors, further decreased survival and proliferation, as well as increased cell death. In summary, both DPP8 and DPP9 activities confine macroautophagy in breast cancer cells. Thus, their inhibition or knockdown reduces cell viability and sensitizes luminal breast cancer cells to tamoxifen treatment.

## 1. Introduction

Proteases are the largest enzyme family in vertebrates, representing about 3% of the human genome [1]. Proteolysis takes place in all cell compartments playing a key role in a variety of cellular processes, such as gene expression, proliferation, differentiation, and cell death. The dipeptidyl-aminopeptidase (DPP) IV family are serine proteases cleaving off dipeptides from the *N*-termini of substrates preferentially after proline [2] or alanine [3,4,5]. The four enzymatically active members of this protease family are called DPP4, fibroblast activation protein-α (FAP-α), DPP8, and DPP9 [2]. DPPIV as well as FAP-α are either soluble or transmembrane proteins, whereas DPP8 and DPP9 are localized in the cytosol, and the long isoform of DPP9 is also able to enter the nucleus [3,6]. DPP8 and DPP9 have a high amino acid sequence similarity of 79% and are conserved across various species [3,7]. Due to their similarity, DPP8 and DPP9 were supposed to cleave identical substrates [2,8]. However, in the last years, unique substrates of DPP8 and DPP9 have been identified [5,9,10,11]. The functions of DPP8 and DPP9 are very diverse, ranging from immunity [5,12,13] and metabolism [2,14] to cancer [15,16,17].

Since 2020, breast cancer represents the most frequent cancer entity worldwide and is still the leading cause of cancer-related deaths among women [18]. Although the outcomes of patients have improved due to better screening methods and individual therapy options, many patients still die due to late diagnosis, aggressive breast cancer subtypes, or acquired tumor resistance towards common therapeutic strategies [19]. Breast cancer is a heterogeneous disorder characterized by the expressions of different receptors, like the estrogen receptor (ER), progesterone receptor (PR), and human epidermal growth factor receptor 2 (HER2, or receptor tyrosine kinase erbB2 (ERBB2)), as well as the proliferation marker Ki-67 [19,20,21]. Thus, breast tumors are divided into at least four different clinically relevant molecular subtypes that determine therapeutic options and patients’ prognoses. Breast tumors with a high expression of ER and/or PR are referred to as luminal tumors, with luminal A tumors (ER^+^, PR^+^, HER2^−^, Ki-67^low^) usually being less proliferative than luminal B tumors (ER^+^, PR^+^, HER2^− or +^, Ki-67^high^). The prognosis of patients with luminal tumors is, in general, good, especially due to the use of endocrine therapy. This type of treatment targets estrogen-dependent cancers via selective ER modulators (SERMs) (tamoxifen) or ER degraders (SERDs) (fulvestrant), or interferes with the production of estrogen via aromatase inhibitors [22,23,24]. HER2-enriched breast cancers (ER^−^, PR^−^, HER2^+^, Ki-67^high^) are associated with an intermediate prognosis, especially due to the use of HER2-targeting antibodies blocking pro-oncogenic signaling [19]. In contrast, basal-like/triple-negative tumors (ER^−^, PR^−^, HER2^−^, Ki-67^high^) are strongly aggressive and therapeutic options are limited to surgery, chemotherapy, and/or radiation.

Although DPP8 and DPP9 have been shown to influence tumor growth in various entities, like Ewing sarcoma [25], cervical cancer [26], or non-small-cell lung cancer [17], their role in breast cancer is unknown. In breast cancer cell lines, similar *DPP8* mRNA and protein levels were detected in MCF-7 (luminal A), MDA.MB-231 (basal-like), and MDA.MB-453 (basal-like) cells [27]. In contrast, the DPP9 protein expression was stronger in the basal-like cell lines compared to MCF-7 cells, whereas the mRNA levels were similar in all cell lines investigated.

In our study, we investigated the impact of DPP8 and DPP9 on breast cancer cells representing different molecular subtypes. For this purpose, we treated MCF-7 (luminal A) and MDA.MB-231 (basal-like) cells with the DPP8/9 inhibitor 1G244. DPP8/9 inhibition led to the accumulation of acidic vesicles, which was more pronounced in MCF-7 cells compared to MDA.MB-231 cells. Further analysis of MCF-7 cells revealed that DPP8/9 inhibition interferes with the formation of LC3B/LAMP1-double-positive autolysosomes and results in the spatial separation of autophagosomes and lysosomes identified via staining for LC3B and LAMP1. Therefore, DPP8/9 inhibition impairs autolysosome formation as an essential step in the process of macroautophagy. In addition, DPP8/9 inhibition reduced cell survival and proliferation, which was further decreased upon a combinatory treatment with tamoxifen. The knockdown of either DPP8 or DPP9 revealed that both proteases contribute to this phenotype.

## 2. Materials and Methods

### 2.1. Cell Culture

Cell culture was generally performed under aseptic conditions in a laminar-flow hood. MCF-7 and MDA.MB-231 cells were obtained from the American Type Culture Collection (ATCC). Before performing the experiments, the identities of the cell lines were confirmed with PCR-based genetic-marker testing by Eurofins Genomics. Cells were cultured in DMEM high glucose supplemented with 10% FCS, 1% L-glutamine, and 1% Penicillin/Streptomycin, and were incubated under controlled conditions at 37 °C and 5% CO_2_. For starvation, the FCS concentration was reduced to 1% FCS. To inhibit DPP8/9, MCF-7 cells were treated with 10 µM 1G244 and MDA.MB-231 cells with 5 µM 1G244 (AK Scientific (Union City, CA, USA), Y0432) or the solvent control DMSO (Sigma-Aldrich, Burlington, MA, USA).

MCF-7 cells were transfected with either the pTCEBAC (Figure S1A) or pTREBAV vector (Figure S1B) containing an shRNA targeting DPP8 or DPP9, respectively, in an miR-E backbone (Table 1). Transfection was previously described in [28]. Shortly, the pMSCV-rtTA3-IRES-EcoReceptor-PGK-Puro vector was integrated using a lentiviral-vector system. Afterwards, ecotropic retroviral transduction was used to integrate pTCEBAC/pTREBAV-single shRNAs into MCF-7 cells. For this purpose, Plat-E cells were transfected with 3.25 μg shRNA and 0.75 μg p-Super-DGCR8 in 500 μL pre-warmed Opti-MEM containing 12 μL polyethylenimine (PEI). The retroviral particles were harvested after 72 h via filtration of the Plat-E supernatant using a disposable syringe and a 0.45 μm sterile filter. The supernatant was diluted 1:6 in DMEM with 8 μg/mL polybrene and incubated with MCF-7 cells for transfection. The next day, the medium was exchanged and selection with blasticidin (26 μg/mL) was started, which was maintained for two weeks. Expression of shRNAs was induced via the addition of 2 µg/mL Doxycycline (Dox).

### 2.2. RNA Isolation and qRT-PCR

RNA was isolated from cells using a Total RNA Kit (peqGOLD) according to the protocol. The RNA concentration was measured via NanoDrop^TM^ 2000 (Thermo Fisher Scientific, Waltham, MA, USA). For RT-PCR, 1 μg RNA was used and transcribed into cDNA using the iScript™ cDNA Synthesis Kit (Bio-Rad, Hercules, CA, USA), according to the protocol. For qPCR, cDNA was mixed with the SYBR^TM^ Select Master Mix (Thermo Fisher Scientific) and forward/reverse primers. Samples were cycled in the CF96 Real-Time machines (Bio-Rad). The following primers were used: *ACTB* forward 5′-AGCACTGTGTTGGCGTACAG-3′ and reverse 5′-CTCTTCCAGCCTTCCTTCCT-3′; *DPP8* forward 5′-GGCCACAAGGATTTACGCAACAAC-3′ and reverse 5′-AAGGTAGCGACTCCAGCTGATCT-3′; *DPP9* forward 5′-TGCAGAAGACGGATGAGTCT-3′ and reverse 5′-GGAATCTCAGAGTAGAGGAG-3′; *LAMP1* forward 5′-TCAGCAGGGGAGAGACACGC-3′ and reverse 5′-CGCTGGCCGAGGTCTTGTTG-3′; *LC3B* forward 5′-ACCATGCCGTCGGAGAAG-3′ and reverse 5′-ATCGTTCTATTATCACCGGGATTTT-3′; *p62* (*SQSTM1*) forward 5′-CCTTTCTGGCCGCTGAGTGC-3′ and reverse 5′-GTCCCCGTCCTCATCCTTTCTCA-3′. Data analysis was performed via the CFX Manager (Bio-Rad).

### 2.3. DPP8/9 Activity Assay

Cells were washed once with DPBS, incubated in 0.05% Trypsin–EDTA for a few minutes at 37 °C, and harvested. After two washing steps with DPBS, cells were resuspended in 150 μL hypotonic buffer (20 mM HEPES at pH 7.9; 1.5 mM MgCl_2_; 10 mM KCl; 0.05% Triton-X-100; and 1 mM DTT in ddH_2_O) and incubated for 10 min on ice before centrifugation at 600 rcf and 4 °C for 6 min. The protein concentration of the supernatant was measured via the Pierce™ BCA Protein Assay Kit (Thermo Fisher Scientific), according to the protocol. An amount of 5 μg protein was mixed with 95 µL hypotonic buffer and 5 μL of 250 µM fluorogenic DPP peptide substrate H-Gly-Pro-AMC (Bachem, Bubendorf, Switzerland). Enzyme activity was measured every minute for 1 h via the EnSpire (Perkin Elmer, Bridgeville, PA, USA) at 480 nm.

### 2.4. LysoTracker^TM^ Staining

Cells were harvested, centrifuged at 300 rcf for 5 min, and washed twice with DPBS. Afterwards, cells were resuspended in FACS buffer (2% FCS and 5 mM EDTA in DPBS) with LysoTracker™ Green DND-26 (Thermo Fisher Scientific; 1:10,000) and incubated for 15 min at 37 °C. Analysis was performed via FACS using the CytoFLEX S (Beckman Coulter, Singapore) and FlowJo 10.6.2 software (BD). For microscopy, cells were seeded on coverslips and stained with LysoTracker™ Green DND-26 diluted in culture medium instead of FACS buffer. In the last 2 min of incubation, Hoechst (1:1000) was added to the medium. Cells were washed once with DPBS, followed by fixation in 4% paraformaldehyde (PFA) for 20 min at RT in the dark. Next, cells were washed once with DPBS, mounted in PermaFluor™ (Thermo Fisher Scientific) on slides, and dried overnight. Analysis was performed with the AxioVert 40C fluorescence microscope (Zeiss, Oberkochen, Germany).

### 2.5. Protein Isolation and Western Blot

Cells were washed three times with pre-chilled DPBS on ice and harvested via scraping in RIPAplus buffer (50 mM Tris-HCl at pH 7.4; 150 mM NaCl; 1 mM EDTA at pH 7; 2.5 mM Na_4_P_2_O_7_; 1 mM β-glycerophosphate; 1% Triton-X-100; 0.001 g/mL SDS; 0.005 g/mL sodium deoxycholate; 1 mM sodium orthovanadate; a PhosSTOP™ tablet/10 mL; and a cOmplete™ ULTRA tablet/10 mL in ddH_2_O). The lysates were incubated for 15 min on ice, vortexed frequently, and mechanically disrupted via Dounce homogenization. After centrifugation at 800 rcf and 4 °C for 15 min, the supernatant was used to determine the protein concentration via the Pierce^TM^ BCA Protein Assay Kit (Thermo Fisher Scientific). The lysates were mixed with 5× protein-loading buffer (250 mM Tris-HCl at pH 6.8; 500 mM DTT; 10% SDS; 0.5% bromophenol blue; 50% glycerol in ddH_2_O) and incubated at 95 °C for 5 min.

The lysates were loaded on a gel and the SDS-PAGE was run at 60 V for 30 min, followed by 120 V for approximately 2 h. Protein transfer onto PVDF membranes was performed via wetblot at 400 mA for about 90 min. For immunoblotting, the membranes were blocked in 3% BSA in 0.1% PBST for 1 h and were afterwards incubated with a primary antibody overnight at RT or 4 °C. Primary antibodies targeting DPP8 (Abcam (Bristol, UK), ab42075, 1:500), DPP9 (Abcam, ab42080, 1:500), LAMP1 (Cell Signaling Technologies (Danvers, MA, USA), 3243, 1:500), LC3B (Cell Signaling Technologies, 2775, 1:500), p62 (SQSTM1: Cell Signaling Technologies, 5114, 1:500), and TUBA (Sigma-Aldrich, T9026, 1:1000) were used. After washing three times with 0.1% PBST for 10 min, membranes were incubated with a secondary antibody for approximately 90 min, followed by three washing steps. As secondary antibodies, Goat-α-Mouse (Sigma-Aldrich, A0168, 1:5000) or Goat-α-Rabbit (Jackson Immuo Research (West Grove, PA, USA), 111-035-003, 1:5000) were used. The membranes were incubated with ECL solution (Thermo Fisher Scientific) for about one minute and afterwards were measured via the VILBER Fusion SL and quantified with FusionCapt Advance software V17.03.

### 2.6. Immunofluorescence

Cells on coverslips were fixed in 4% PFA for 20 min and washed once with DPBS. After additional washing with PBST for 10 min, cells were permeabilized via −20 °C methanol for 10 min, followed by 0.2% Saponin in PBST for another 5 min. Next, cells were washed three times with PBST for 10 min and blocked with 0.1% Saponin in 1% BSA in PBST for 20 min. After a further washing step, primary antibodies were added and incubated overnight at 4 °C. Primary antibodies targeting LAMP1 (Abcam, ab25245, 1:750), LC3B (Cell Signaling Technologies, 2775, 1:500), KIF5B (Thermo Fisher Scientific, PA1-643, 1:250), and Dynein-1 (Invitrogen (Carlsbad, CA, USA), MA1-070, 1:250) were used. Cells were washed three times with PBST for 10 min. The secondary antibodies were added and incubated in the dark at RT for two hours and, in the last two minutes of incubation, Hoechst (1:1000) was added. As secondary antibodies, Goat-α-Rat Alexa 555 (Invitrogen, A-21434, 1:1000) or Donkey-α-Rabbit Alexa 488 (Invitrogen, A-11001, 1:1000) were used. After a last washing step with PBST, coverslips were mounted in PermaFluor™ on slides and dried overnight. Analysis was performed with the LSM 710 confocal laser scanning microscope (Zeiss).

### 2.7. β-Galactosidase Staining

Cells on coverslips were stained for β-galactosidase using the Senescence β-Galactosidase Staining Kit (Cell Signaling Technology), according to the protocol. After staining was completed, cells were mounted in PermaFluor™ on slides and dried overnight. Analysis was performed with the BZ-9000 microscope (Keyence, Singapore).

### 2.8. Plate Colony-Formation Assay

For colony formation, 1400 cells/well were seeded in 6-well plates and incubated at 37 °C and 5% CO_2_. One day after seeding, 5 μM 4-hydroxytamoxifen (4-OHT) was added and refreshed every two days for 15 days. After 15 days of culture, cells were rinsed once with DPBS and stained with 1% crystal violet in 20% methanol for 10 min. Cells were washed thoroughly with tap water and air-dried overnight. Pictures were taken using a light desk and the Canon PowerShot G6 camera (Canon, Tokyo, Japan). Analysis was performed with the ImageJ plugin Colony Area according to the protocol in [29].

### 2.9. Trypan Blue Staining

Cells were treated with 10 μM or 15 μM 4-OHT for 48 h. Medium was collected, and cells were detached and transferred in the same Falcon tube. Cells were centrifuged at 300 rcf for 5 min, cell pellets were resuspended in 1 mL medium, and 10 μL was mixed 1:1 with trypan blue to stain dead cells. Stained cells were pipetted into a Neubauer counting chamber and analyzed using a microscope (Nikon, Tokyo, Japan).

### 2.10. FACS

To analyze the fluorescent reporters of pTCEBAC and pTREBAV, cells were detached from the culture dish after Doxycycline treatment and centrifuged at 300 rcf for 5 min. After centrifugation, cells were washed twice with DPBS and resuspended in FACS buffer. Measurement was performed with the LSR II (BD) and BD FACS Diva 6.1.2 software. Data analysis was performed with FlowJo 10.6.2 software.

### 2.11. Data Presentation and Statistics

Unless stated otherwise, the data of independent experiments are presented as means + standard errors of the means (SEMs). Statistical analyses were carried out with OriginPro 2020 (OriginLab). The statistical significance of the difference of the means between two groups was analyzed via paired-sample *t*-test.

## 3. Results

### 3.1. Inhibition of DPP8/9 Increases Acidic Endolysosomal Compartment in Different Breast Cancer Cell Lines

**Figure 1:**
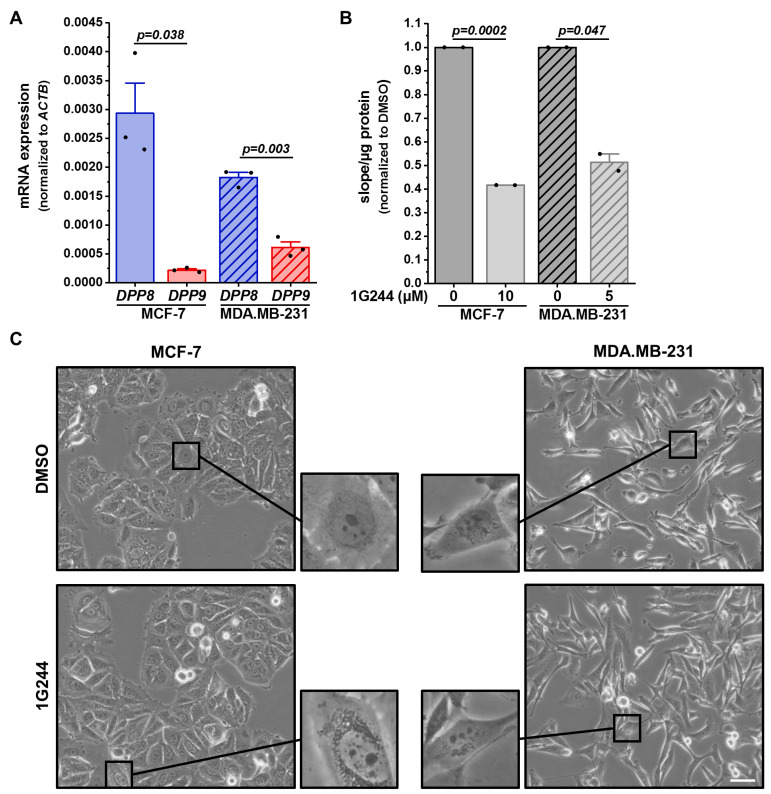
DPP8/9 inhibition leads to accumulation of vesicles in human breast cancer cells. (**A**) Relative *DPP8* (blue) and *DPP9* (red) mRNA expressions normalized to *ACTB* in MCF-7 and MDA.MB-231 cells via qRT-PCR (*n* = 3). (**B**) Cleavage of DPP8/9 substrate H-Gly-Pro-AMC in MCF-7 and MDA.MB-231 cells treated with 1G244 or solvent control DMSO for 48 h (*n* = 2). (**C**) Representative pictures of MCF-7 and MDA.MB-231 cells treated with 1G244 or solvent control DMSO for 48 h (*n* = 3). Scale bar: 50 μm. Bar charts show all data points with means + SEMs and *p*-values calculated via paired-sample *t*-test.

To investigate the role of DPP8 and DPP9 in human breast cancer cells, MCF-7 (luminal A) and MDA.MB-231 (basal-like) cells were characterized concerning their *DPP8* and *DPP9* mRNA expressions (Figure 1A). MCF-7 cells showed higher *DPP8* mRNA levels compared to MDA.MB-231 cells, whereas the *DPP9* mRNA was higher expressed in the basal-like cells than in the luminal A cells. Interestingly, *DPP8* was higher expressed in both cell lines compared to *DPP9*. To address the functions of DPP8 and DPP9 in both cell lines, cells were treated with the combined DPP8/9 inhibitor 1G244, as no selective inhibitor of only one of these proteases is available [2]. DMSO treatment was used as the solvent control. The inhibition was measured via an aminopeptidase activity assay utilizing the fluorogenic peptide H-Gly-Pro-AMC. The inhibitor treatment reduced the cleavage of the peptide by about 60% in MCF-7 cells and 50% in MDA.MB-231 cells (Figure 1B). Notably, observing the cells under the microscope revealed that DPP8/DPP9 inhibition led to the accumulation of vesicles in the cytoplasm of both cell lines (Figure 1C). This phenotype was more pronounced in the MCF-7 cells compared to the MDA.MB-231 cells.

**Figure 2:**
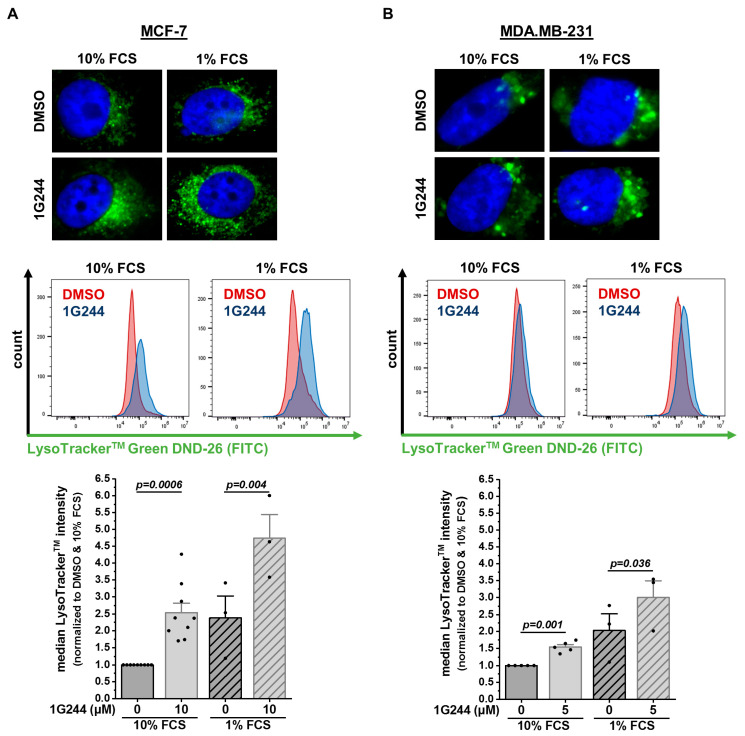
DPP8/9 inhibition leads to accumulation of LysoTracker^TM^-positive vesicles in human breast cancer cells. (**A**,**B**) Fluorescence microscopy and flow cytometry analysis of LysoTracker™ Green DND-26 staining of (**A**) MCF-7 and (**B**) MDA.MB-231 cells ± 1G244 and 10% or 1% FCS (*n* ≥ 3). Bar charts show all data points with means + SEMs and *p*-values calculated via paired-sample *t*-test.

To investigate whether the observed accumulation of vesicles originated from acidic cell compartments, like lysosomes or other organelles fused with lysosomes [30], cells were stained with LysoTracker™ Green DND-26. In MCF-7 cells, the DPP8/9 inhibition more than doubled the compartment stained with LysoTracker™ Green DND-26 compared to the solvent control (Figure 2A). Additionally, starvation using only 1% FCS instead of 10% FCS further enhanced the vesicles stained by LysoTracker™ Green DND-26 in the DMSO- and 1G244-treated MCF-7 cells but was again higher upon DPP8/9 inhibition. In the MDA.MB-231 cells, treatment with 1G244 also elevated the stained vesicles compared to DMSO under nutrient-rich and -deprived conditions (Figure 2B). However, the effect in these basal-like breast cancer cells was weaker than in MCF-7 cells. Thus, DPP8/9 inhibition led to the accumulation of acidic vesicles in the cytosols of breast cancer cells, and this effect was most prominent in the luminal MCF-7 cells. Therefore, the following experiments focused on the nature of these vesicles in MCF-7 cells.

### 3.2. DPP8/9 Inhibition Dysregulates Lysosomal Positioning and Thereby Autophagy in Luminal Breast Cancer Cells

**Figure 3:**
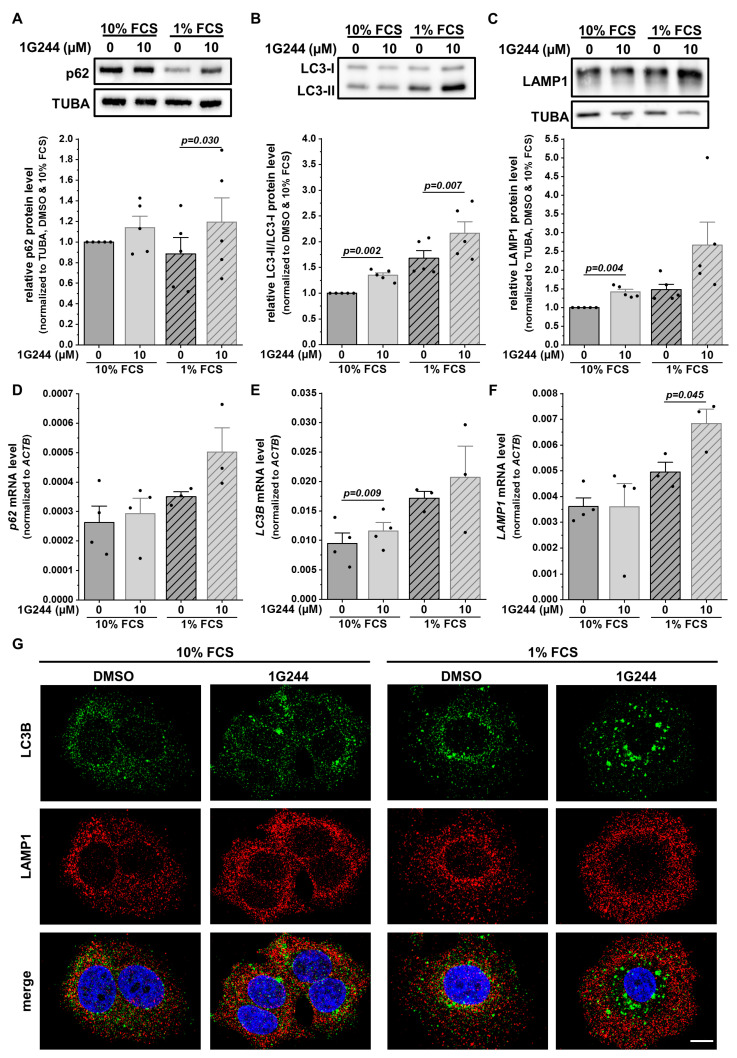
DPP8/9 inhibition separates lysosomes and autophagosomes in MCF-7 cells. (**A**–**C**) (**A**) p62/SQSTM1, (**B**) LC3-I/LC3-II, and (**C**) LAMP1 protein expressions in MCF-7 cells ± 1G244 and 10% or 1% FCS via Western blot. Protein expressions of p62/SQSTM1 and LAMP1 were normalized to TUBA (*n* = 5). (**D**–**F**) Relative (**D**) *p62*/*SQSTM1*, (**E**) *LC3B*, and (**F**) *LAMP1* mRNA expressions normalized to *ACTB* in MCF-7 cells ± 1G244 and 10% or 1% FCS via qRT-PCR (*n* ≥ 3). (**G**) Immunofluorescence of LC3B (green) and LAMP1 (red) in MCF-7 cells ± 1G244 and 10% or 1% FCS (DNA: Hoechst (blue); *n* = 3). Scale bar: 10 μm. Bar charts show all data points with means + SEMs and *p*-values calculated via paired-sample *t*-test.

Lysosomal storage disorders showing similar accumulations of lysosomes present mainly with a defect in autophagy [31]. Thus, proteins involved in the process of macroautophagy were analyzed. The cargo receptor p62 (SQSTM1) can bind misfolded proteins or dysfunctional organelles intended for autophagic degradation [32]. Hence, p62 protein levels can be used as a marker for autophagic flux, accumulating upon autophagy inhibition. In MCF-7 cells, the protein levels of p62 increased due to 1G244 treatment, with only slight changes upon starvation (Figure 3A). To analyze the autophagosome formation, the conversion of the autophagosomal membrane precursor LC3-I into LC3-II [33] was measured, revealing a higher LC3-I/LC3-II ratio upon DPP8/9 inhibition (Figure 3B). This difference was also detected due to the starvation of the cells with higher baseline levels compared to the use of 10% FCS medium. For the degradation of the engulfed cargo, autophagosomes need to fuse with lysosomes providing hydrolases for proteolysis to form autolysosomes [30]. The levels of the lysosomal membrane protein LAMP1 were also elevated upon 1G244 treatment, especially upon 1% compared to 10% FCS (Figure 3C).

Interestingly, not all observed changes at the protein level were also detected at the mRNA level, especially under nutrient-rich conditions. The levels of *p62* (Figure 3D) and *LAMP1* (Figure 3F) stayed similar despite the use of 1G244, whereas a slight but significant increase in the *LC3B* mRNA expression was detected due to DPP8/9 inhibition (Figure 3E). However, serum starvation increased the mRNA levels of all the analyzed markers, which was stronger for the 1G244-treated cells compared to the DMSO treatment.

For further investigation of the autophagic process, MCF-7 cells were stained with antibodies targeting LC3B (autophagosomes: green) and LAMP1 (lysosomes: red). Fluorescence microscopy revealed the single-stained puncta of both proteins under nutrient-rich conditions with a higher abundance in the DPP8/9-inhibited cells compared to the controls (Figure 3G). Some structures were stained for both markers used (yellow) in all conditions, indicating the formation of autolysosomes. FCS starvation elevated the occurrence of LC3B- and LAMP1-double-positive puncta independent of 1G244 treatment. In the DMSO control cells, the general increase in staining was accompanied by several double stainings. Interestingly, the inhibition of DPP8/9 under starvation conditions shifted the spatial localization of LAMP1 staining from the perinuclear region to the periphery of the cell, reducing the occurrence of LC3B- and LAMP1-double-positive puncta. Thus, although the inhibition of DPP8/9 elevated the protein levels of p62, LAMP1, as well as the LC3-I/LC3-II ratio, indicating the induction of macroautophagy, the observed spatial separation of autophagosomes and lysosomes upon DPP8/9 inhibition indicates an impaired autophagosome–lysosome fusion and, as a consequence, compromised autophagic flux.

### 3.3. Inhibition of DPP8/9 Results in Nucelar Localization of Vesicle Transport Protein KIF5B

**Figure 4:**
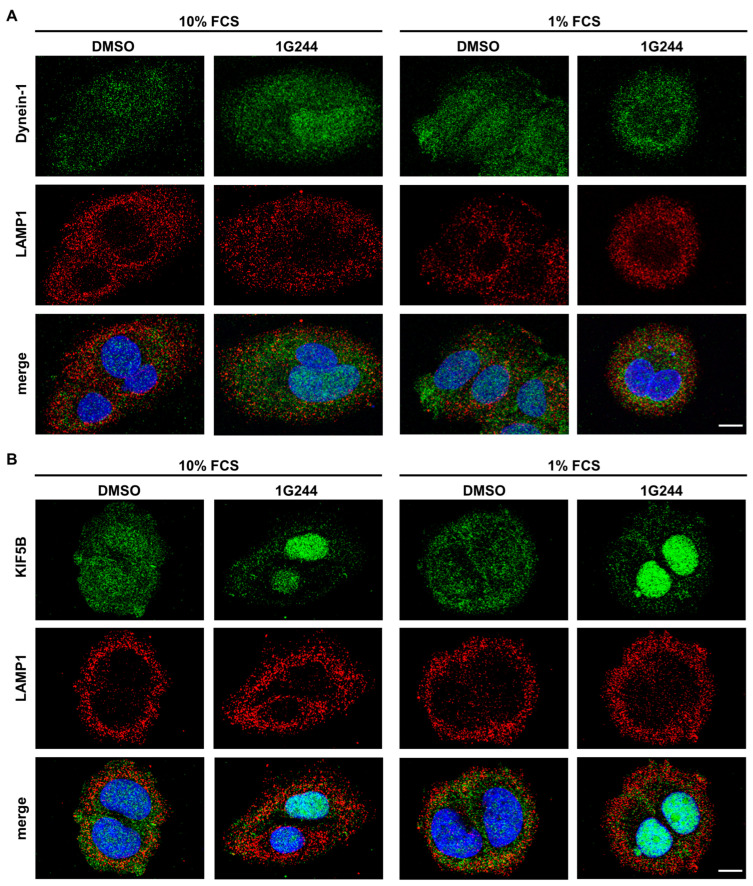
DPP8/9 inhibition localizes KIF5B in the nuclei of MCF-7 cells. (**A**,**B**) Immunofluorescence of (**A**) Dynein-1 or (**B**) KIF5B (green) and LAMP1 (red) in MCF-7 cells ± 1G244 and 10% or 1% FCS (DNA: Hoechst (blue); *n* = 3). Scale bar: 10 μm.

The observed change in the localization of the lysosomes to the periphery of the cell and, as a consequence, the spatial separation from autophagosomes may be caused by defective vesicular transport interfering with autophagosome–lysosome fusion. Here, the Dynein-Dynactin motor complex is mainly responsible for vesicle transport to the perinuclear region (minus end) along microtubules, whereas kinesins facilitate the movement of vesicles to the cell periphery (plus end) [34]. To investigate whether these processes are disturbed upon DPP8/9 inhibition, MCF-7 cells were stained with antibodies targeting either Dynein-1 (green; Figure 4A), responsible for minus-end transport, or kinesin-1 (KIF5B: green; Figure 4B), facilitating plus-end transport, both together with LAMP1 (red). Dynein-1 was dispersed in the whole cell independent of the use of the DPP8/9 inhibitor 1G244 or serum starvation. Furthermore, some overlap of Dynein-1 and LAMP1 staining was observed showing yellow puncta, indicating no impairment of perinuclear transport. Staining of KIF5B showed a similar distribution as Dynein-1 for the controls under nutrient-rich as well as -starved conditions. However, DPP8/9 inhibition by 1G244 resulted in an increase in the nuclear staining of KIF5B compared to the controls, independent of the culture conditions. Due to the mainly nuclear localization of KIF5B and the already above-described peripheral distribution of LAMP1-positive vesicles, the occurrence of double-positive structures was considerably less than in the controls. These results indicate that DPP8/9 is involved in maintaining the localization of KIF5B in the cytoplasm, which is important for accurate vesicle trafficking, and therefore contributes to efficient autophagy.

### 3.4. Knockdown of Either DPP8 or DPP9 Enhances Autophagy in Luminal Breast Cancer Cells

To investigate the contribution of DPP8 and DPP9 to the regulation of autophagy, MCF-7 cells were transfected with Doxycycline (Dox)-inducible vectors containing an shRNA targeting either DPP8 or DPP9. The induction efficacy was measured via FACS analysis of the fluorescent vectors (pTCEBAC + *shDPP8*: Figure S1C; pTREBAV + *shDPP9*: Figure S1D), showing more than 85% fluorescent cells. The Doxycycline-induced knockdown of either DPP8 or DPP9 was verified at the mRNA level (*DPP8*: Figure S1E; *DPP9*: Figure S1F) and protein level (DPP8: Figure S1G; DPP9: Figure S1H), and via the enzyme activity (Figure S1I). Notably, the knockdown of DPP8 or DPP9 did not affect the mRNA expressions of *DPP9* or *DPP8*, respectively.

**Figure 5:**
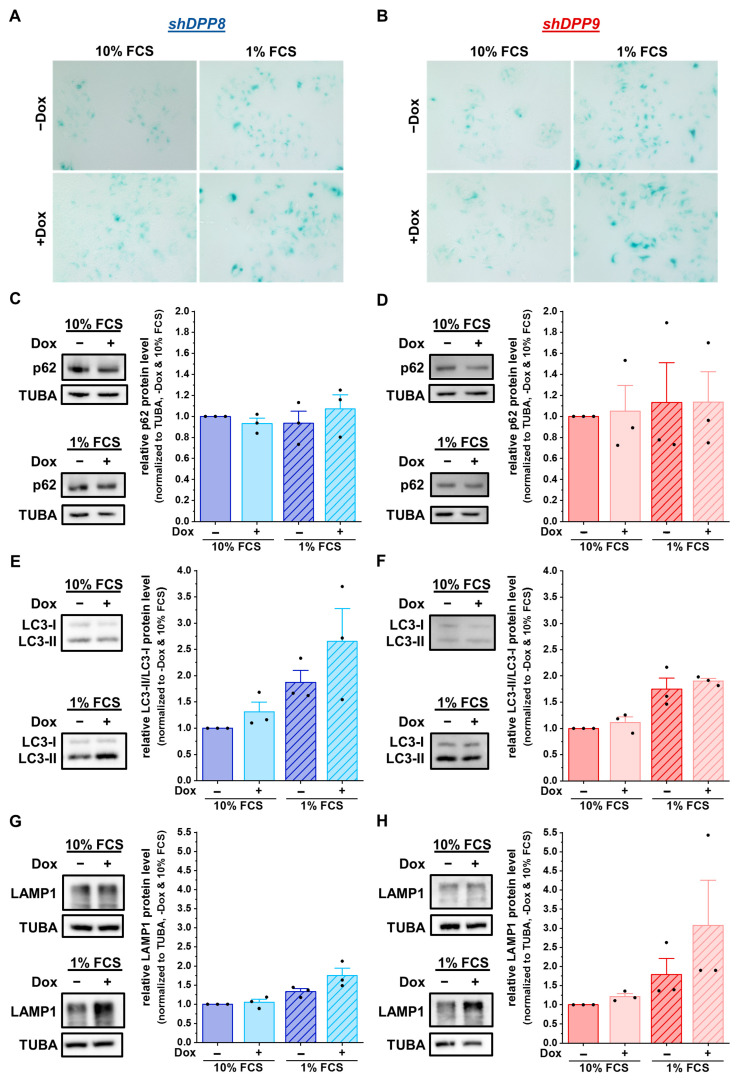
DPP8 or DPP9 knockdown enhances autophagy in MCF-7 cells. (**A**,**B**) Staining of β-galactosidase in *shDPP8*- (**A**) or *shDPP9*-transduced MCF-7 cells (**B**) ± Dox and 10% or 1% FCS (n = 3). (**C**–**H**) p62/SQSTM1 (**C**,**D**), LC3-I/LC3-II (E,F), and LAMP1 (**G**,**H**) protein expression in *shDPP8*- (**C**,**E**,**G**) or *shDPP9*-transduced MCF-7 cells (**D**,**F**,**H**) ± Dox and 10% or 1% FCS by Western blot. Protein expression of p62/SQSTM1 and LAMP1 was normalized to TUBA (*n* = 3). Bar charts show all data points with mean + SEM.

The effect of a single DPP8 or DPP9 knockdown on autophagy was first analyzed via staining for β-galactosidase, which is localized in lysosomes and can be used to visualize them. Serum starvation increased the β-galactosidase staining in MCF-7 cells. Interestingly, *shDPP8*- (Figure 5A) as well as *shDPP9*-mediated knockdown (Figure 5B) further increased the acidic β-galactosidase staining compared to the control cells (Dox) under nutrient-rich and, especially, nutrient-reduced conditions.

To further examine the autophagy-related effects caused by the knockdown of DPP8 or DPP9 in MCF-7 cells, the protein levels of the autophagy markers p62 and LC3 were assessed. The levels of p62 remained constant in all conditions (*shDPP8*: Figure 5C; *shDPP9*: Figure 5D). In contrast, the ratio of LC3-II/LC3-I increased upon the knockdown of DPP8, showing a further elevation due to the use of 1% FCS medium (Figure 5E). This effect was not observed upon the knockdown of DPP9 (Figure 5F). Analysis of the LAMP1 levels showed no difference upon the Dox-induced *shDPP8*-mediated knockdown with 10% FCS medium and only a slight increase upon starvation compared to uninduced cells (Figure 5G). Conversely, DPP9 knockdown increased the LAMP1 levels under starved conditions (Figure 5H). Further analysis of the mRNA expression showed no difference upon DPP8 knockdown (*p62*/*SQSTM1*: Figure S2A; *LC3B*: Figure S2B; *LAMP1*: Figure S2C) or DPP9 knockdown compared to the controls (*p62*/*SQSTM1*: Figure S2D; *LC3B*: Figure S2E; *LAMP1*: Figure S2F). These results demonstrate that DPP8 and DPP9 knockdown increased the acidic compartment independent from each other, but their impacts on autophagy and the compartments contributing to this process seem to be different.

### 3.5. Inhibition and Single Knockdown of DPP8/9 Increases Responsiveness of Luminal Breast Cancer Cells to Tamoxifen

**Figure 6:**
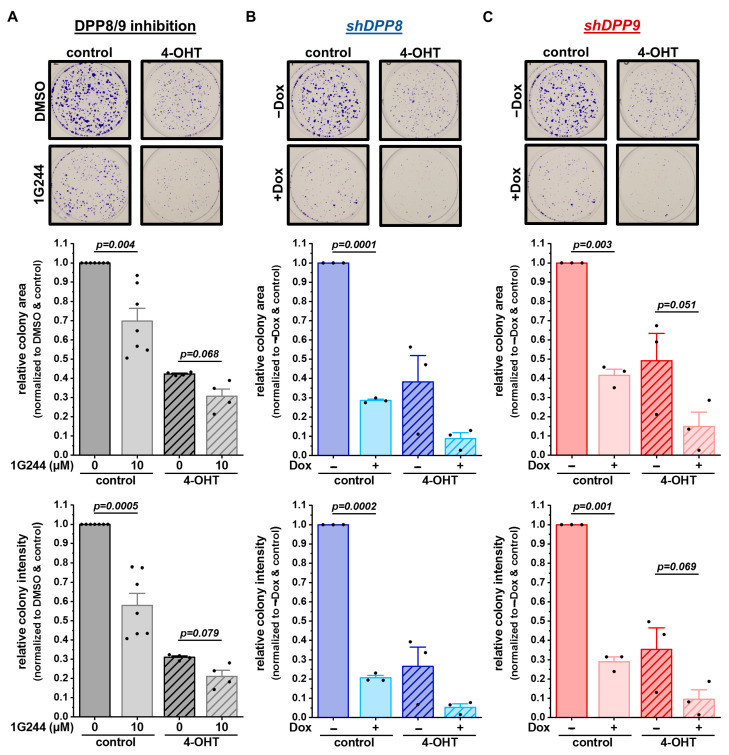
DPP8/9 inhibition, DPP8 or DPP9 knockdown in combination with 4-OHT treatment reduces survival and proliferation of MCF-7 cells. (**A**–**C**) Representative pictures of colonies stained by crystal violet and quantitative analysis of colony area and intensity of (**A**) DPP8/9-inhibited (±1G244) and (**B**) *shDPP8*- or (**C**) *shDPP9*-transduced MCF-7 cells ± Dox and ± 5 µM 4-OHT (*n* ≥ 3). Bar charts show all data points with means + SEMs and *p*-values calculated via paired-sample *t*-test.

Impaired autophagy is known to affect tumor cell proliferation, growth, and survival [35]. Thus, the survival of MCF-7 cells upon DPP8/9 inhibition by 1G244 or the shRNA-mediated knockdown of DPP8 or DPP9 was assessed via colony-formation assays. Additionally, 4-hydroxytamoxifen (4-OHT), the active metabolite of the SERM tamoxifen, which is a standard therapy for breast cancer patients with luminal subtypes [23], was used. DPP8/9 inhibition significantly reduced cell proliferation and survival, indicated by the respective reductions in the colony area and colony intensity (Figure 6A). The use of 5 µM of 4-OHT alone decreased the colony area and intensity to about 40% compared to the controls, with a further significant reduction upon additional 1G244 treatment. In line with these observations, *shDPP8*- (Figure 6B) or *shDPP9*-mediated knockdown (Figure 6C) significantly reduced the colony area and colony intensity to levels comparable to the single 4-OHT treatment. The combination of knockdown and 4-OHT further decreased the colony area/intensity to about 10%/5% for *shDPP8* and 15%/10% for *shDPP9*.

**Figure 7:**
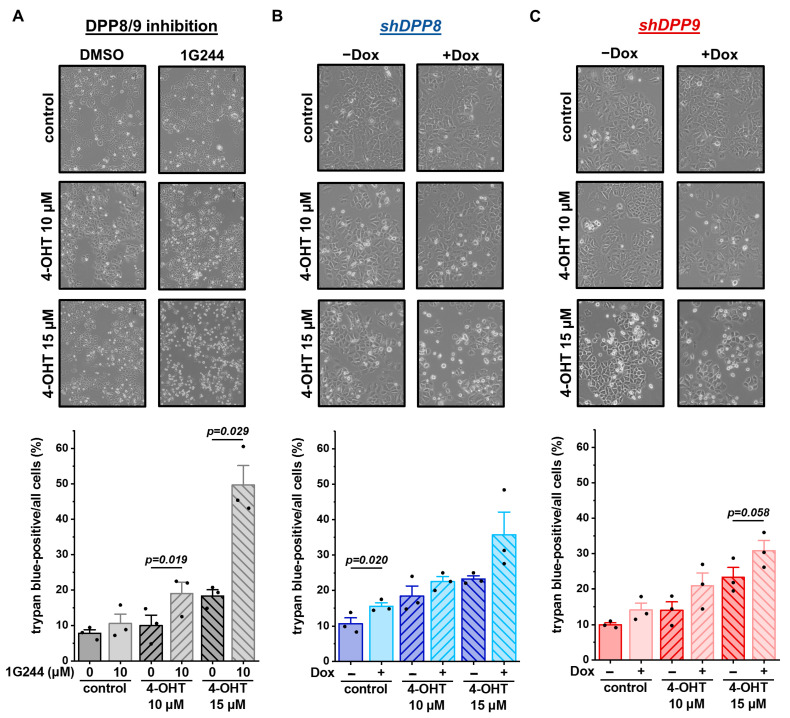
DPP8/9 inhibition, DPP8 or DPP9 knockdown in combination with 4-OHT increases cell death of MCF-7 cells. (**A**–**C**) Representative pictures of MCF-7 cells and quantitative analysis of trypan-blue-positive cells of (**A**) DPP8/9-inhibited (±1G244) and (**B**) *shDPP8*- or (**C**) *shDPP9*-transduced MCF-7 cells ± Dox and ±10 µM or 15 µM 4-OHT (*n* = 3). Bar charts show all data points with means + SEMs and *p*-values calculated via paired-sample *t*-test.

To elucidate whether these different treatments not only affect cell survival and proliferation, but also cell death, cells were stained with trypan blue. The treatment of MCF-7 cells with 1G244 only mildly affected cell death, leading to a slightly increased number of dead cells in the culture (Figure 7A). The addition of 10 µM or 15 µM 4-OHT induced cell death in a concentration-dependent manner. Interestingly, the combination of the 1G244 and 4-OHT treatments significantly increased cell death and at least doubled the number of dead cells compared to the 4-OHT or 1G244 treatments alone. A similar trend was also measured for the *shDPP8*-mediated (Figure 7B) and *shDPP9*-mediated (Figure 7C) knockdown in the MCF-7 cells. However, the effect of the knockdown with the 15 µM 4-OHT treatment was not as strong as for the use of 1G244, inhibiting both DPP8 and DPP9. Thus, DPP8/9 inhibition increased the sensitivity of luminal MCF-7 cells to 4-OHT treatment by reducing cell survival and proliferation and enhancing cell death.

## 4. Discussion

Our results demonstrate that DPP8 and DPP9 play a pivotal role in starvation-induced autophagy, especially during the transport of autophagosomes and lysosomes to different subcellular localizations, as shown by the immunofluorescence labeling of these compartments. Upon autophagy activation [36], newly formed autophagosomes are transported to the perinuclear region to fuse with lysosomes [37,38]. In MDA.MB-231 cells representing basal-like breast cancer, an increase in LysoTracker^TM^-positive vesicles was measured upon DPP8/9 inhibition. However, the inhibition of DPP8/9 in MCF-7 cells (luminal A) resulted in an even stronger accumulation of lysosomes, especially in the peripheries of the cells, and less of the motor protein KIF5B in the cytoplasm. Although kinesins like KIF5B are reported to transport vesicles to the cell periphery, the deficiency of KIF5B in HeLa cells results in the spatial separation of autophagosomes, being predominantly in the perinuclear region, and lysosomes distributed in the periphery of the cell [39]. This phenotype is identical to our findings upon DPP8/9 inhibition—especially in serum-starved MCF-7 cells—indicating that the mainly nuclear localization of KIF5B upon DPP8/9 inhibition in our experiments may be the cause of this spatial separation. Furthermore, murine KIF5B was identified in a screen searching for DPP9 substrates [40]. Therefore, the effect of DPP8 and DPP9 on the localization of KIF5B may be direct due to the cleavage of KIF5B by these enzymes.

This defective lysosomal positioning due to DPP8/9 inhibition seems to cause the measured increase in vesicles belonging to the endolysosomal compartment, especially upon serum starvation, when cells are highly dependent on autophagy [36,41]. Because the correct intracellular positioning of autophagosomes and lysosomes is critical for their fusion during autophagy, interference in this process strongly influences autophagic flux [37,38]. The knockdown of either DPP8 or DPP9 increased acidic vesicles, although DPP8 seems to have a higher impact on autophagosomes, whereas DPP9 mainly affects the lysosomal compartment. This demonstrates that both proteases regulate autophagy, and a deficiency in either DPP8 or DPP9 results in a similar phenotype of dysfunctional autophagy. However, their functions in this degradation pathway are likely to differ from each other, requiring further investigations.

The impact of autophagy on tumor development and progression is ambiguous [35,42]. Physiologically, autophagy maintains homeostasis in cells, thereby preventing tumor development via the degradation of dysfunctional organelles and misfolded or oncogenic proteins. In contrast, in established cancers, autophagy can be either tumor-promoting or -suppressive. Nevertheless, due to its importance in hypoxic [35,43] or nutrient-deprived conditions [35,44], which are especially present in proliferating tumors, most studies focus on reducing autophagy in cancer cells. Thus, inhibiting DPP8/9 may be a potential therapeutic approach to reduce proliferation and enhance cell death, as reported here for the MCF-7 cells.

DPP8 and DPP9 are reported to affect proliferation as well as cell death in several ways. The knockdown of DPP9 in oral squamous cell carcinoma cells increased cell growth via FAP-α [45], whereas the overexpression of DPP9 in Huh-7 and HepG2 cells reduced proliferation and increased apoptosis independent of its enzymatic activity by interacting with HRAS [46]. In contrast, DPP9 deficiency in non-small-cell lung cancer cells [17], as well as DPP8 deficiency in cervical cancer cells, reduced proliferation [26]. In Ewing Sarcoma Family of Tumor (ESFT) cells, the knockdown of DPP4, DPP8, or DPP9 leads to NPY-mediated apoptosis [25]. Therefore, the effect of DPP8 and DPP9 on tumor cell proliferation and apoptosis seems to be cell-type-specific.

About 70% of all breast malignancies are ER-positive breast cancers, in which mainly the oncogenic ER-signaling pathway promotes malignant cell proliferation and tumor growth [19,47]. Targeting ER signaling via endocrine therapy has substantially reduced breast cancer mortality in the past decades. The dominating agent used in endocrine therapy is tamoxifen, a SERM that interacts with the ER and inhibits the transcription of estrogen-responsive target genes in breast cancer cells. The treatment of MCF-7 cells with 4-OHT significantly reduced cell growth and enhanced cell death, demonstrating the dependence of luminal A breast cancer cells on ER signaling.

However, resistance to endocrine therapy is a major clinical problem. Approximately one-third of ER-positive breast tumors develop resistance to endocrine therapy, worsening the prognoses of many breast cancer patients [48,49]. Several underlying resistance mechanisms affecting different oncogenic pathways have been elucidated in the last years, including the loss of the ER, alterations in tyrosine kinase signaling, the deregulation of the cell cycle, or the aberrant expressions of apoptosis regulators. Furthermore, autophagy has been identified as a crucial cellular pathway, mediating therapy resistance in multiple types of cancer [50,51,52]. It is known that tamoxifen treatment induces autophagy-mediated endocrine resistance in ER-positive breast cancer cells [52], but the mechanism resulting in the resistance of these cells is still poorly understood. Nevertheless, a recent study showed that tamoxifen affects the lysosome integrity and induces lysosomal damage in these cells [51]. Interestingly, ER-positive breast cancer cells resistant to endocrine therapy presented with an increased autophagic flux and were less susceptible to lysosomal damage induced by tamoxifen. The inhibition of autophagy by, for instance, chloroquine re-sensitized tamoxifen-resistant breast cancer cells to endocrine therapy [51,52]. Because our data show that DPP8/9 inhibition can increase the sensitivity of luminal MCF-7 cells to 4-OHT treatment, significantly reducing cell survival and proliferation and enhancing cell death, targeting DPP8 and DPP9 in combination with anti-estrogen therapy may be a potential treatment strategy for ER-positive breast cancers. This treatment strategy might improve the response to endocrine therapeutics, delay or prevent the development of endocrine therapy resistance, and/or restore sensitivity towards anti-estrogen therapeutics in resistant tumor cells.

## 5. Conclusions

In this study, we show that DPP8 and DPP9 play a pivotal role in maintaining autophagic flux and thereby contribute to the better survival of ER-/PR-positive breast tumor cells (luminal A). This seems to be strongly dependent on vesicle transport, and especially the localization of the motor protein KIF5B, although the mechanism as to how DPP8 and/or DPP9 influence its localization within the cell remains unclear. Furthermore, combinatory treatment using 4-OHT (SERM) and 1G244 (DPP8/9 inhibitor) reduced proliferation as well as enhanced cell death, demonstrating its potency in ER-positive breast cancer cells. Nevertheless, further investigation of breast cancer cell lines representing different molecular subtypes of breast cancer, as well as other cancer entities, is necessary to see whether this phenotype is cell-type-specific or transferable to other cell types.

## Figures and Tables

**Table 1:** Sequence of shRNAs in miR-E backbone.

Target	Sequence (5′–3′)
DPP8	TGCTGTTGACAGTGAGCGCACGGTTTGTGGTAGTAATCTATAGTGAAGCCACAGATGTATAGATTACTACCACAAACCGTATGCCTACTGCCTCGGA
DPP9	TGCTGTTGACAGTGAGCGCCCACGGCTTCCTGGACGAAAATAGTGAAGCCACAGATGTATTTTCGTCCAGGAAGCCGTGGATGCCTACTGCCTCGGA

## Data Availability

The data generated in this study are available within the article, its supplementary data files, and upon request via contact with the corresponding author.

## Supplementary Materials

The following supporting information can be downloaded at https://www.mdpi.com/article/10.3390/cells12162031/s1, Figure S1: Validation of DPP8 or DPP9 knockdown in MCF-7 cells; Figure S2: Expression of autophagosomal and lysosomal marker upon DPP8 or DPP9 knockdown in MCF-7 cells.
