# Supplementary material for: Dipeptidyl-Aminopeptidases 8 and 9 Regulate Autophagy and Tamoxifen Response in Breast Cancer Cells
Source: Cells. 2023 Aug 10;12(16):2031. doi: 10.3390/cells12162031 (PMC10453625; doi:10.3390/cells12162031)
Supplement: Supplementary file 1 [file cells-12-02031-s001.zip › cells-2488338-supplementary.pdf]

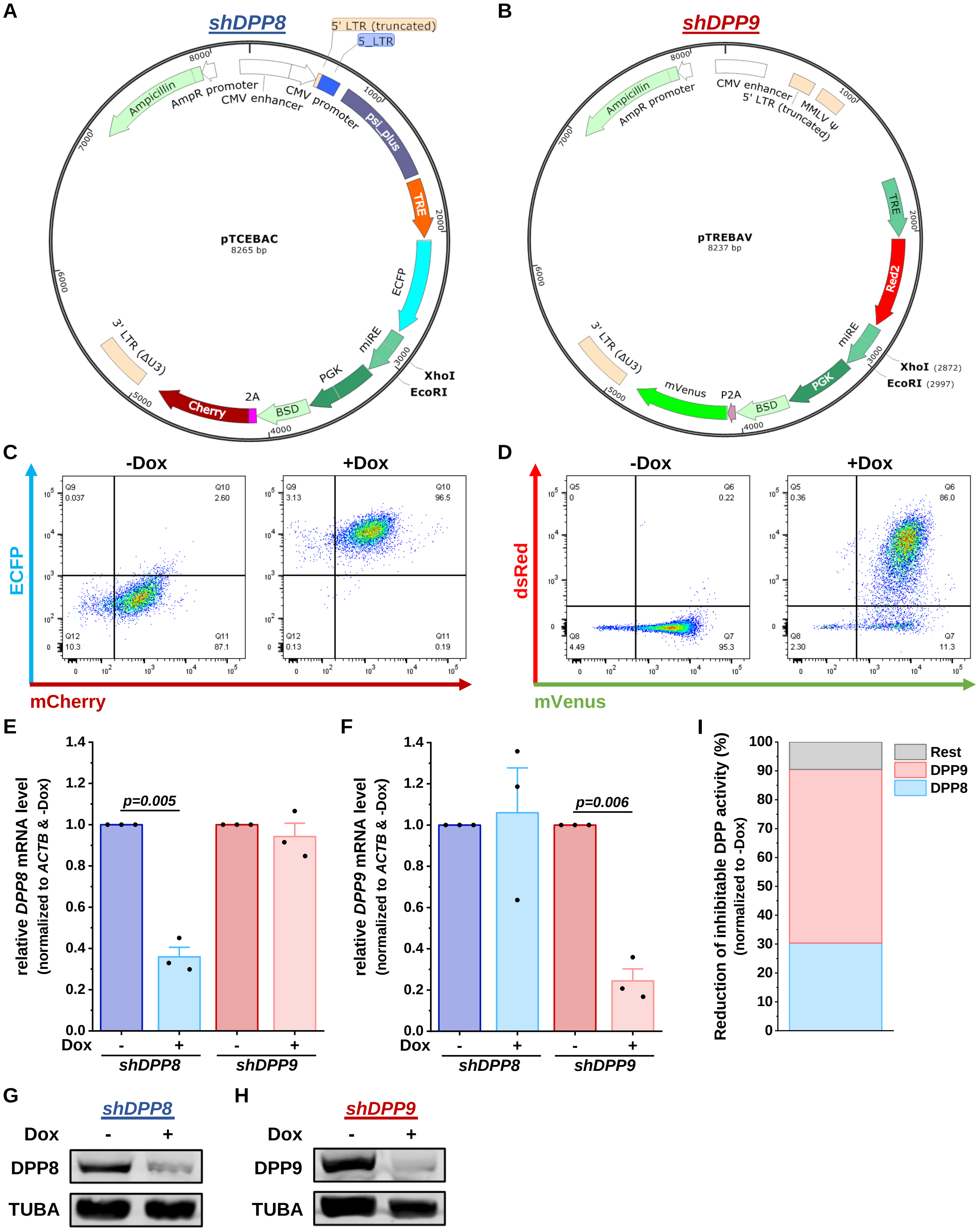


**Figure S1: Validation of DPP8 or DPP9 knockdown in MCF-7 cells. (A)** pTCEBAC with contistuive mCherry and inducible ECFP fluorescence and shDPP8. **(B)** pTREBAV with constitutive mVenus and inducible dsRed fluorescence and shDPP9. **(C-D)** FACS of MCF-7 cells ± Dox with pTCEBAC + shDPP8 **(C)** or pTREBAV + shDPP9 **(D)**. **(E-F)** Relative *DPP8* **(E)** and *DPP9* **(F)** mRNA expression normalized to *ACTB*, in *shDPP8*- or *shDPP9*-transduced MCF-7 cells ± Dox by qRT-PCR (n=3). **(G-H)** DPP8 **(G)** and DPP9 **(H)** with TUBA protein levels of *shDPP8*- or *shDPP9*-transduced MCF-7 cells ± Dox by Western Blot. **(I)** Relative reduction of inhibitable DPP activity in *shDPP8*- or *shDPP9*-transduced MCF-7 cells (n=3). Bar charts show all data points with mean + SEM and *p*-value calculated by paired-sample t-test.

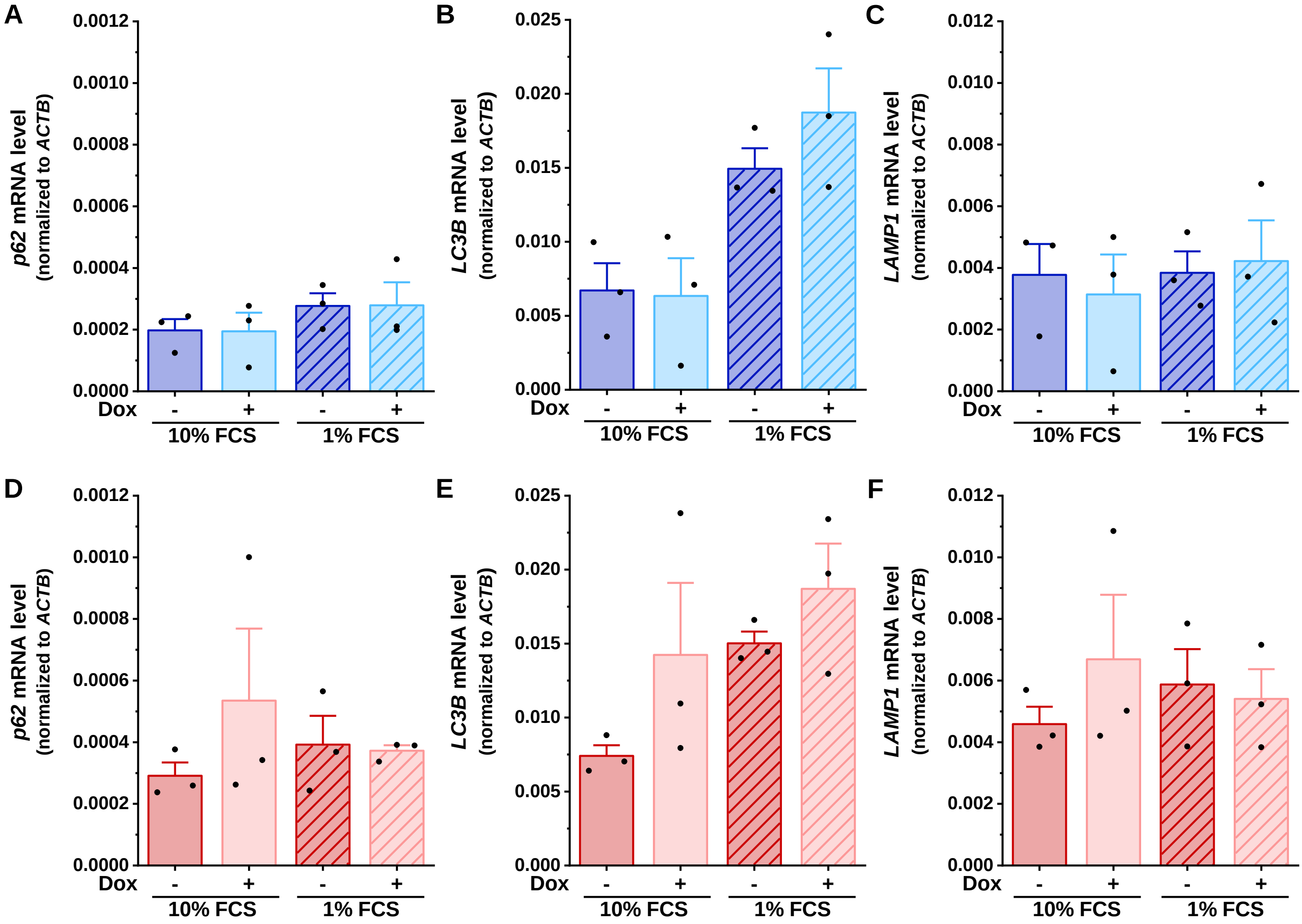


**Figure S2: Expression of autophagosomal and lysosomal marker upon DPP8 or DPP9 knockdown in MCF-7 cells. (A-C)** Relative *p62/SQSTM1* **(A)**, *LC3B* **(B)**, and *LAMP1* **(C)** mRNA expression normalized to *ACTB* in *shDPP8*-transduced MCF-7 cells ± Dox and 10% or 1% FCS by qRT-PCR (n=3). **(D-F)** Relative *p62/SQSTM1* **(D)**, *LC3B* **(E)**, and *LAMP1* **(F)** mRNA expression normalized to *ACTB* in *shDPP9*-transduced MCF-7 cells ± Dox and 10% or 1% FCS by qRT-PCR (n=3). Bar charts show all data points with mean + SEM.
